# Transcriptome Profiling of Goose Ovarian Follicle Granulosa Cells Reveals Key Regulatory Networks for Follicle Selection

**DOI:** 10.3390/ani13132132

**Published:** 2023-06-28

**Authors:** Jie Liu, Shudi Dai, Zichun Dai, Yuyan Feng, Mingming Lei, Rong Chen, Huanxi Zhu

**Affiliations:** 1 Institute of Animal Science, Jiangsu Academy of Agricultural Sciences, Nanjing 210014, China; liujie891213@163.com (J.L.); 19599965308@163.com (Z.D.); 20140036@jaas.ac.cn (M.L.); 2 Key Laboratory of Crop and Livestock Integration, Ministry of Agriculture, Nanjing 210014, China; 3 School of Life Science, Jiangsu University, Zhenjiang 212000, China; 20210064@jaas.ac.cn; 4 Key Laboratory of Animal Physiology & Biochemistry, Nanjing Agricultural University, Nanjing 210095, China; jinpengwu123021@gmail.com

**Keywords:** goose, follicle selection, granulosa cell, transcriptome profiling, constructing, ceRNA network

## Abstract

**Simple Summary:**

The follicular selection stage during the development of goose follicles is a decisive step in egg production, but current research is still incomplete. With the help of high-throughput sequencing technology, we analyzed the mRNAs, miRNAs, and lncRNAs that play a critical role only in the follicular selection stage and ultimately constructed a ceRNA regulatory network. We confirmed the significant changes in the extracellular matrix, cell junction, and glucose and lipid metabolism during follicular selection. More importantly, using a new analytical approach, we identified miRNAs that play an important role in follicle selection, including miR-222-3p, miR-2954-3p, miR-126-5p, miR-2478, and miR-425-5p. This study provides potential regulatory targets for precise regulation of goose production performance.

**Abstract:**

The selection of follicles determines the reproductive performance of birds, but the process of follicle selection in geese is still elusive. This study focuses on Yangzhou geese during the egg-laying period and divides the follicular development process into three stages: small follicle development, follicle selection, and follicle maturation. Transcriptome sequencing was performed on granulosa cells from large white follicles, small yellow follicles, and F5 and F4 follicles. In addition, we selected the transcripts that remained unchanged during the development and maturation of small follicles but significantly changed during the follicular selection stage as the transcript collection that plays an important role in the follicular selection process. Then, we performed functional analysis on these transcripts and constructed a ceRNA network. The results showed that during the follicular selection stage, the number of differentially expressed mRNAs, miRNAs, and lncRNAs was the highest. In addition, miR-222-3p, miR-2954-3p, miR-126-5p, miR-2478, and miR-425-5p are potential key core regulatory molecules in the selection stage of goose follicles. These results can provide a reference for a better understanding of the basic mechanisms of the goose follicle selection process and potential targets for the precise regulation of goose egg production performance.

## 1. Introduction

Geese lack systematic breeding and have relatively low reproductive efficiency compared to chickens [1]. There are variations between breeds, but most geese produce only tens of eggs annually. As a result, low egg production has hindered the rapid development of the goose industry. Follicle development in poultry is an important factor determining egg-laying traits. During the non-breeding season, there are primarily small white follicles and few large white follicles in the ovaries of poultry [2]. During the breeding season, small yellow follicles appear in the ovaries and undergo selection to form hierarchical follicles that enter the ovulation sequence. The number and size of hierarchical follicles in chickens is well-documented [3]. However, research on the follicular development and selection process in goose breeds remains incomplete. Follicle selection is a decisive step that determines the number of hierarchical follicles and the eventual number of eggs laid [4]. Therefore, clarifying the molecular mechanisms underlying follicle selection has important theoretical and practical significance for farming and animal husbandry.

High-throughput genetic sequencing has accelerated research on the developmental process of goose follicles, but existing research has focused on comparing the differences between different breeding stages of the same goose variety [5,6,7]. During this process, researchers have discovered many differential transcripts and proposed multiple signaling pathways that may be related to follicular development and egg production. However, transcriptional maps (mRNA and miRNA) of goose egg follicle development were not reported in geese until 2019 [8]. Research has shown that connection, adhesion, and lipid metabolism play crucial roles in follicular selection. These results were based on cluster analysis of transcripts with two expression patterns, namely transcripts with similar expression levels in 4–6 mm to 8–10 mm follicles, but with significant changes in 8–10 mm to F5 follicles. This analysis method excludes transcripts involved in the development of pre-hierarchical follicles but does not exclude important transcripts and signaling pathways in the development of hierarchical follicles. During the development of hierarchical follicles, yolk material accumulates, resulting in the largest change in follicle volume and a darkening of color. At this stage, contraction of the actin cytoskeleton and vascular smooth muscle changes significantly [9]. However, these processes are not core events in follicle selection. Therefore, new analytical approaches to identify transcripts and signaling pathways involved only in the follicular selection stage should be established.

Follicular selection is a “one-plank bridge” in the fate of follicles. Most follicles undergo follicular atresia due to apoptosis of granulosa cells [10,11], while only a few pre-hierarchical follicles can pass through this bridge to become hierarchical follicles. The state of granulosa cells plays a decisive role in the fate of follicles. Li et al., conducted mRNA and miRNA expression profiling analysis on granulosa cells and theca cells of the same goose during the egg-laying period [8]. The number of differentially expressed transcripts was counted, and the results showed that during the follicular selection stage, the number of mRNAs changed in granulosa cells was 2782 but only 415 in theca cells. Similarly, during the follicular selection stage, the number of miRNAs varied in granulosa cells was 117 but only 54 in theca cells. This result suggests that granulosa cells play a more important role in goose follicular selection compared to membrane cells. Therefore, this study selected follicular granulosa cells as the research object. Firstly, we accurately classified female goose follicles during the laying period, taking into account the color, diameter, and vascular richness of the follicles. On this basis, large white follicles (LWFs), small yellow follicles (SYFs), and F5 and F4 follicles were selected as the research subjects. And the follicular development process from LWFs to F4 was subdivided into three stages: small follicle development (LWFs to SYFs), follicle selection (SYFs to F5), and follicle maturation (F5 to F4). The transcripts that remained unchanged in the LWFs to SYFs and F5 to F4 stages but exhibited significant changes between the SYFs to F5 stage were identified as transcripts important for goose follicle selection. Subsequently, enrichment analysis was conducted on the target transcript, and the ceRNA regulatory network was constructed by selecting the five miRNAs with the highest expression abundance from the target transcript library. These results can provide a reference for a better understanding of the basic mechanism of the goose follicle selection process and provide potential targets for precise regulation of goose egg production performance.

## 2. Materials and Methods

### 2.1. Ethical Approval

The experimental procedures were approved by the Research Committee of the Jiangsu Academy of Agricultural Sciences and were conducted in accordance with the Regulations for the Administration of Affairs Concerning Experimental Animals (Decree No. 63 of the Jiangsu Academy of Agricultural Science on 8 July 2014).

### 2.2. Animal Resources and Sample Collection

**Figure 1:**
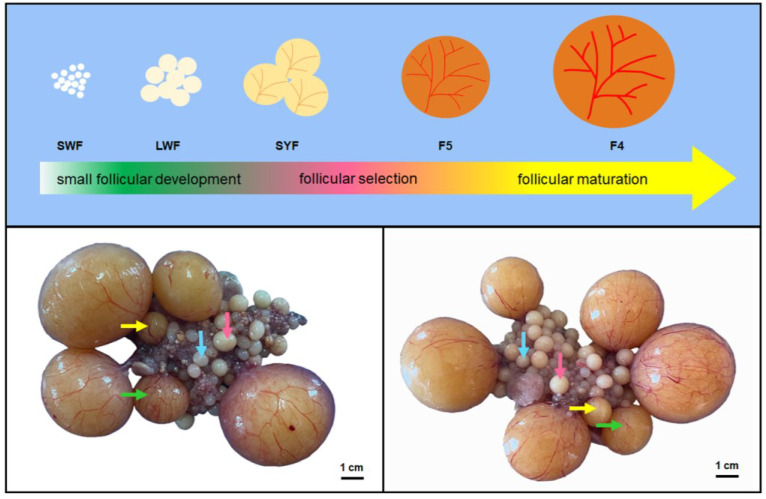
Schematic diagram of follicular development and sampling of Yangzhou geese ovarian follicles. The process of follicles from SWFs to ovulation can be divided into three stages: small follicular development, follicular selection, and follicular maturation. The follicle cohorts include pre-hierarchical (SWFs, LWFs, and SYFs) and hierarchical follicles (F5, F4, F3, F2, and F1). We collected LWFs, SYFs, and F5 and F4 follicles for experimental research. LWFs: the follicles are milky white with a diameter of 4–6 mm (blue arrow). SYFs: the follicles are milky yellow in color, with a diameter generally greater than 8 mm, and there are thin blood vessels on the surface of the follicles (pink arrow). F5: the follicles are orange in color, with a diameter greater than 12 mm, and numerous thick blood vessels distributed on the surface (yellow arrow). F4: the follicles are orange in color with a diameter greater than F5, and there are numerous thick blood vessels distributed on the surface (green arrow).

The experiment was conducted in March. Yangzhou geese of the same batch and source, aged 42 weeks, were selected as the research subjects. Due to the frequency of laying eggs for female geese being 1 egg per 2 days, we took measures to control the follicular development status of the female geese. On the afternoon before the sampling day, we gently pressed the lower abdomen of the goose to confirm if there were hard shell eggs in the abdomen. Only female geese with hard shell eggs in their lower abdomen could be selected for sampling the next day. A total of nine geese were selected, and follicles were extracted and separated from the nine geese. The follicle identification and classification criteria were based on the diameter, appearance, and vascular distribution of the follicles (Figure 1). LWFs are milky white with a diameter of 4–6 mm. SYFs are milky yellow in color, with a diameter generally greater than 8 mm and thin blood vessels on their surface. The F5 follicles are orange in color, with a diameter greater than 12 mm. Numerous thick blood vessels were distributed on the surface of the follicles; the F4 follicles were orange in color with a diameter greater than that of the F5 follicles, and there were numerous thick blood vessels distributed on the surface of the follicles. Our sampling time lasted from 6:00 a.m. to 10:30 a.m. The follicle diameter was measured by a Vernier scale, especially the diameter of F1–F5. F1 has a diameter of 49.34 ± 2.09 mm, F2 has a diameter of 43.66 ± 2.23 mm, F3 has a diameter of 36.65 ± 3.09 mm, F4 has a diameter of 29.50 ± 2.79 mm, and F5 has a diameter of 19.69 ± 3.65 mm.

The steps for separating the F4 and F5 granulosa cells are as follows: after stripping the connective tissue on the surface of the follicle, place the follicle in a dish containing PBS; use tweezers to tear open the follicles and squeeze out a portion of the yolk; gently shake the plate and remove the membrane layer (theca cell) along the edge of the follicle opening; gently remove the transparent granulosa layer covering the yolk with elbow tweezers; and rinse the attached yolk substance in PBS and quickly freeze it for storage. The steps of separating SYF and LWF granulosa cells are as follows: after the connective tissue on the follicle surface is stripped, place the follicle in a dish containing PBS; use tweezers to tear open the follicles and gently shake them by gripping the edges; and rinse the granulosa layer and freeze it quickly.

Nine samples were divided into three groups for high-throughput sequencing.

### 2.3. RNA Isolation, Library Construction, and Sequencing

Total RNA was extracted from the samples using TRIzol reagent (Invitrogen, Waltham, MA, USA). Total RNA purity was checked using a NanoPhotometer^®^ (München, Germany). RNA concentration was checked using a Qubit^®^ (Dorset, UK) spectrophotometer, and RNA integrity was checked using a Bioanalyzer 2100 system. Three replicate samples were processed for each follicular stage, and 12 libraries were sequenced. The mRNA and small RNA libraries were sequenced by Genedenovo Biotechnology Co. (Guangzhou, China) using Illumina HiSeqTM 4000 and Illumina HiSeq Xten, respectively.

### 2.4. Targets Prediction and Enrichment Analysis

Raw reads were first processed, and reads containing adapters, lay-N, and low-quality reads were removed. The Q20, Q30, and GC contents of the cleaned data were calculated. All downstream analyses were based on the cleaned data. The clean reads were mapped to the Anser cygnoides genome (GCF_000971095.1).

The mRNAs, miRNAs, lncRNAs, and partial circRNA were analyzed. Trend analysis was performed for all transcripts (LWF-SYF-F5-F4). First, based on these results, transcripts with similar expression levels in the LWF to SYF and F5 to F4 stages but with significant changes in SYF to F5 were selected. Venn analysis was performed on the differential transcripts of LWFs vs. SYFs, SYFs vs. F5, and F5 vs. F4, selecting the transcripts that showed significant changes only in SYFs vs. F5. Finally, based on trend and Venn analyses, a transcript set that only changed during the follicular selection stage was obtained for subsequent enrichment analysis. KEGG enrichment analyses were performed using the R package based on hypergeometric distributions. Analysis was performed using OmicShare tools, a free online platform for data analysis (https://www.omicshare.com/tools (accessed on 1 February 2023)).

### 2.5. Construction of the lncRNA-miRNA-mRNA Regulatory Network

To determine the interactions among the differentially expressed (DE) mRNAs, lncRNAs, circRNAs, and miRNAs, we constructed a circRNA/lncRNA-miRNA-mRNA regulatory network based on the ceRNA hypothesis. Miranda and Targetscan were used to predict miRNA-lncRNA, miRNA-mRNA, and miRNA-circRNA pairs, and the correlation between these pairs was evaluated using the Spearman correlation coefficient according to their expression. The interaction network was displayed using Cytoscape software (version 11.0.13, Washington, DC, USA).

### 2.6. Confirmation of the Expression Level of mRNAs, miRNAs, and lncRNAs

Real-time PCR was performed to validate the expression levels of mRNAs, lncRNAs, and miRNAs; three mRNAs, three lncRNAs, and three miRNAs were selected.

Total RNA was isolated from granulosa cells using 0.5 mL TRIzol reagent (Vazyme, Nanjing, China). RNA was reverse transcribed into cDNA using an RT Reagent Kit (Vazyme, Nanjing, China). Approximately 1 μg of cDNA was used for the real-time PCR. Primers for mRNA and lncRNAs were designed using Primer 3 (online). Technical variations were normalized using γ-DH as the endogenous control for lncRNA and mRNA and U6 as the internal reference for miRNA. Primers for mRNAs and lncRNAs were designed using Primer 3 (online) and synthesized by Tsingke Biotechnology Co., Ltd. (Table S2). miRNA quantification was determined by using Bulge-Loop™ miRNA qRT-PCR Primer Sets (one RT primer and a pair of qRT-PCR primers for each set) specific to miR-222-3p, miR-126-5p, and miR-2954-3p, designed by RiboBio (RiboBio, Guangzhou, China). The real-time PCR results were analyzed using the 2^−ΔΔCT^ method, and the abundance of mRNA was expressed as the fold change relative to the mean value of the female group with a short photoperiod.

## 3. Results

### 3.1. mRNA, miRNA, and lncRNA Transcriptomes of Geese Granulosa Cells

After stringent filtering, approximately 15.3 Gb of clean reads were generated per sample by Illumina HiSeqTM 4000 sequencing. These clean reads were mapped to the *Anser cygnoides* genome (GCF_000971095.1), and the percentages of total and uniquely mapped mRNA were greater than 86.68% and 84.32%, respectively (Table S1). We conducted a comparative analysis of lncRNAs based on the reference genome using HISAT2 software. In addition, the clean tags were aligned with the reference genome and then searched against the miRBase database to identify known miRNAs. According to their genome positions and hairpin structures predicted by software mirdeep2, the novel miRNA candidates were identified.

**Figure 2:**
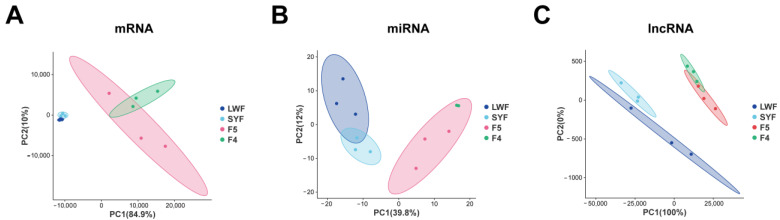
Principal component analysis of messenger RNAs (mRNAs) (**A**), microRNAs (miRNAs) (**B**), and long noncoding RNAs (lncRNAs) (**C**) transcriptome sequencing of granulosa cells. Follicles at the follicular development stage (LWFs to SYFs) or the follicular maturation stage (F5 to F4) are similar. The follicles in the follicular selection stage (SYFs to F5) have significant differences.

Principal component analysis (PCA) was performed on the normalized gene expression data. According to the results, there was significant separation between pre-hierarchical and hierarchical follicles and a significant difference between SYFs and F5 (Figure 2).

**Figure 3:**
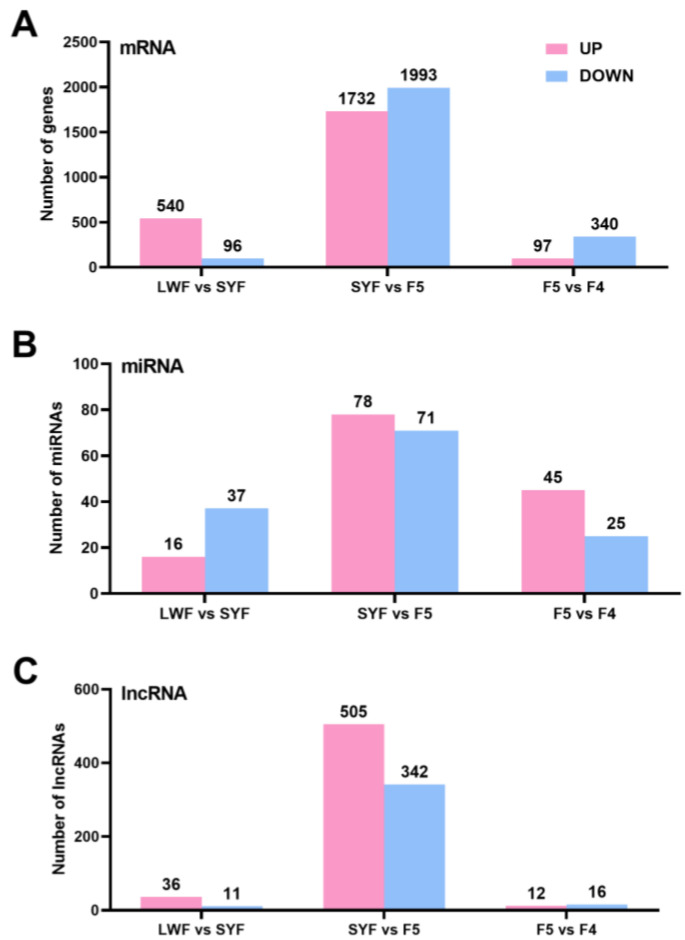
Number of upregulated or downregulated differentially expressed genes (DEGs) in granulosa cells: messenger RNAs (mRNAs) (**A**), microRNAs (miRNAs) (**B**), and long noncoding RNAs (lncRNAs) (**C**).

Based on the results of the DE analysis, we screened the mRNAs and lncRNAs with FDR < 0.05 and |log2FC| > 1 as significant DE mRNAs or lncRNAs. miRNAs with a *p* < 0.05 and a change in their expression level of more than two times were considered DE miRNAs. Figure 3 shows the number of differentially ex-ressed genes (DEGs) among the three groups. During the follicular selection stage, there were significant changes in the expression levels of 3725 mRNAs, 149 miRNAs, and 847 lncRNAs in granulosa cells (Figure 3).

### 3.2. Identification of Transcripts Involved in Follicle Selection in Granulosa Cells

**Figure 4:**
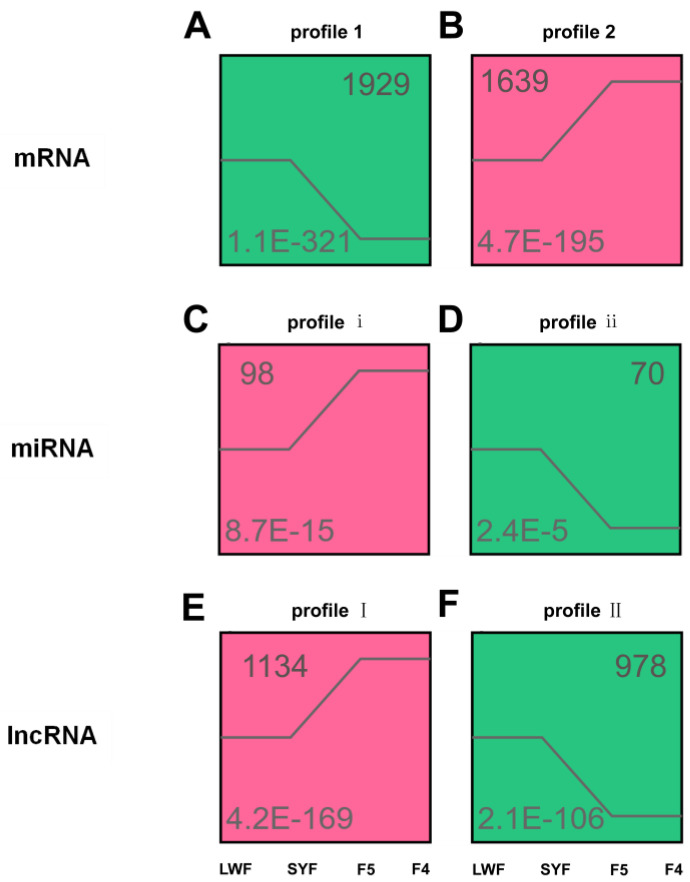
Time-series cluster analysis for the messenger RNAs (mRNAs), microRNAs (miRNAs), and long noncoding RNAs (lncRNAs) in the granulosa cells. Two expression patterns are displayed for mRNA (**A**,**B**), miRNA (**C**,**D**), and lncRNA (**E**,**F**).

To further narrow down the list of target transcripts that may be involved in follicle selection, STEM analysis was performed on the DEGs (LWF-SYF-F5-F4). Among all of the expression patterns, the expression levels that remained unchanged before follicular selection (LWF-SYF), increased or decreased during follicular selection (SYF-F5), and remained unchanged after follicular selection (F5-F4) were selected (Figure 4). All six selected expression patterns showed significant changes and served as important bases for determining the transcripts involved in follicular selection.

**Figure 5:**
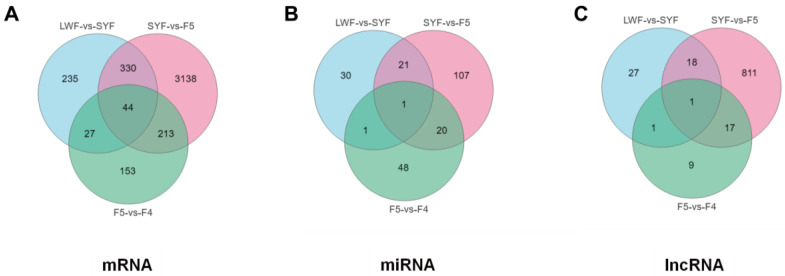
Differentially expressed genes (DEGs) of granulosa cells stratified by messenger RNAs (mRNAs) (**A**), microRNAs (miRNAs) (**B**), and long noncoding RNAs (lncRNAs) (**C**) from three comparison groups.

Venn analysis was performed on the DE-mRNA, miRNA, and lncRNA of the three comparative groups, and transcripts with significant differences observed only in the SYF-F5 stage were selected based on the results (Figure 5).

Finally, we screened the target transcripts to ensure that they existed in both the sets obtained from the trend analysis and the sets obtained from the Venn analysis. This part of the transcript is believed to play an important role in follicular selection, including 2371 mRNAs, 71 miRNAs, and 602 lncRNAs.

### 3.3. Enrichment Analysis of the Selected mRNAs, miRNAs, and lncRNAs

**Figure 6:**
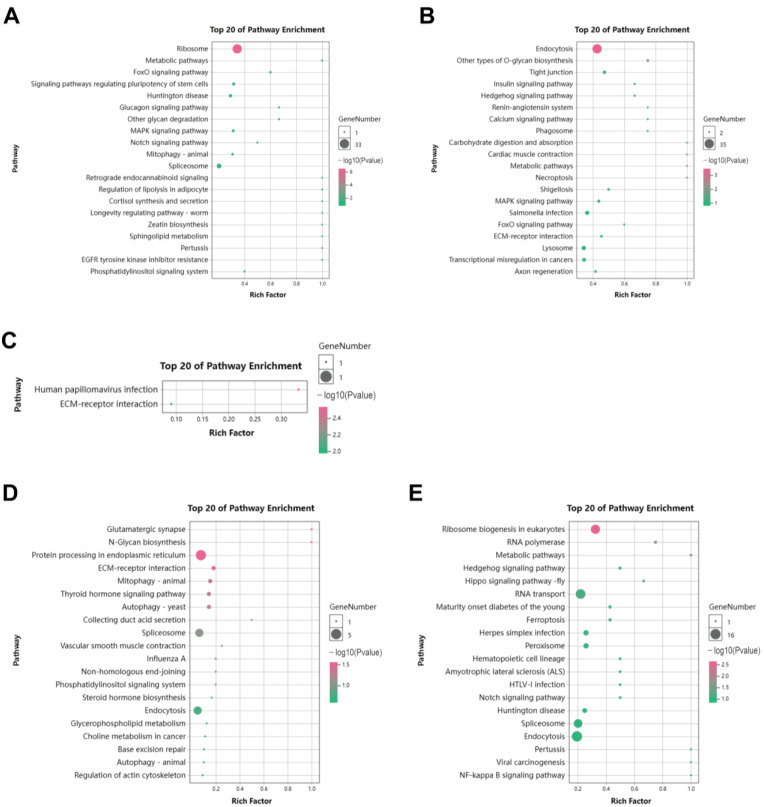
Kyoto Encyclopedia of Genes and Genomes (KEGG) pathways of DE-transcripts in granulosa cells: (**A**) top 20 KEGG pathways of DE-mRNAs; (**B**) top 20 KEGG pathways of DE-miRNAs; (**C**–**E**) top 20 KEGG pathways of antisense, cis, and trans lncRNAs.

Enrichment analysis was conducted on mRNAs, miRNAs, and lncRNAs that play important roles in the follicular selection stage. mRNAs were directly enriched for analysis (Figure 6A). miRNA target genes were predicted using miRanda software, followed by KEGG enrichment analysis of the target genes (Figure 6B). Additionally, the target genes of the selected lncRNAs were predicted to have three regulatory modes: resistance, cis, and trans. Enrichment analysis was performed on the three target mRNA components (Figure 6C–E). The results showed that the transcripts playing an important role in follicular selection were mainly enriched for the extracellular matrix, cell junctions, glucose and lipid metabolism, and signal transduction pathways.

### 3.4. Construction and Analysis of ceRNAs Regulatory Network

**Figure 7:**
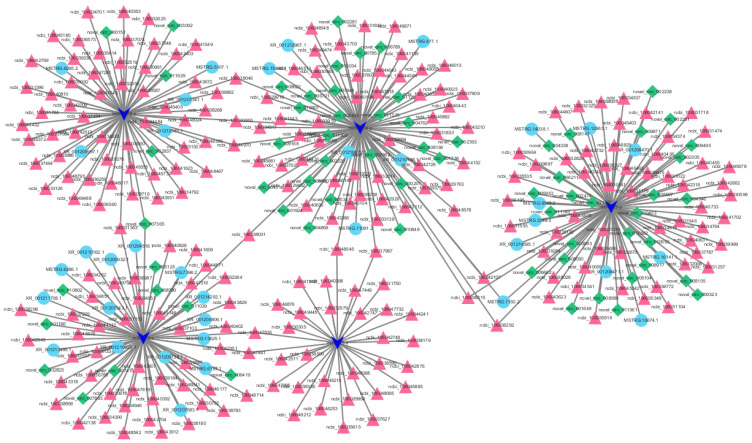
A predicted ceRNA network based on miR-222-3p, miR-2954-3p, miR-126-5p, miR-2478, and miR-425-5p. Dark blue arrows, pink triangles, light blue circles, and green squares represent miRNA, mRNA, lncRNA, and circRNA, respectively.

Competitive endogenous RNAs (ceRNAs) are present in cells, and ceRNA molecules (mRNA, lncRNA, pseudogenes, etc.) can compete to bind the same miRNAs through miRNA response elements (MREs) to regulate their expression levels. miRNAs form the core of ceRNA networks. Based on their expression levels, we selected high-abundance miR-222-3p, miR-2954-3p, miR-126-5p, miR-2478, and miR-425-5p from the 71 selected miRNAs to construct a ceRNA network (Figure 7). 

The miR-222-3p, miR-2954-3p, miR-126-5p, miR-2478, and miR-425-5p contained 95, 90, 73, 68, and 32 ceRNAs, respectively. Among these, the ceRNA molecules targeted by miR-425-5p contained only mRNAs. Additionally, the connectivity of mRNAs such as ncbi_106031363 (RAB3), ncbi_106033555 (Pigq), ncbi_106038031 (EPHA7), ncbi_106037967 (BSDC1), and ncbi_106042107 (SERINC5) was slightly higher.

### 3.5. Real-time PCR Validation of DE-mRNAs, DE-lncRNAs, and DE-miRNAs

**Figure 8:**
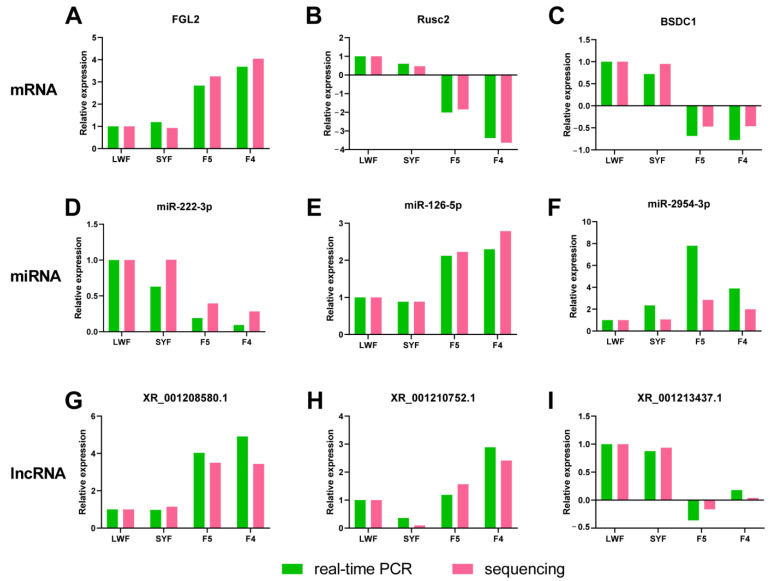
Confirmation of messenger RNAs (mRNAs) (**A**–**C**), microRNAs (miRNAs) (**D**–**F**), and long noncoding RNAs (lncRNAs) (**G**–**I**) in granulosa cells by real-time PCR.

To validate the expression patterns of mRNAs and lncRNAs, qRT-PCR was used to detect the expression of the six selected DEGs. Transcript selection was mainly based on expression patterns. As shown in Figure 8A–C,G–I, all six randomly selected transcripts showed similar expression patterns between qRT-PCR and Illumina sequencing, although there were slight differences in the fold change values.

As shown in Figure 8D–F, there was a similarity between the quantitative assay and high-throughput sequencing analysis of the three miRNAs in terms of fold change of expression. Although there were few differences in the fold changes of the expression of miR-222-3p and miR-126-5p, the variation trends of the three miRNAs were identical.

## 4. Discussion

The purpose of this study is to identify transcripts, especially miRNAs, that play an important role in the follicular selection stage, rather than in the pre-hierarchical follicular development stage and the hierarchical follicular maturation stage. The results of Tianfu meat geese showed that compared to the theca cells of follicles, granulosa cells play a more important role in follicular development [8]. Therefore, we selected granulosa cells from different developmental stages of Yangzhou goose ovaries during the egg-laying period for RNA-sequencing studies.

Significant differences among goose breeds make it difficult to standardize the classification of goose follicles. One study classified white follicles as LWFs with a diameter greater than 6 mm in Zhedong white geese, yellow follicles as SYFs with a diameter of 10–20 mm, and yellow follicles as hierarchical follicles with a diameter greater than 20 mm [12]. Another study reported that white follicles with a diameter of less than 6 mm in Magang geese were LWFs, yellow follicles with a diameter of less than 8 mm were SYFs, and yellow follicles were hierarchical follicles with a diameter of more than 9 mm [13]. Tianfu meat geese have 8–10 mm follicles that have been classified as SYFs [8]. During follicle selection, the vascular network of the follicle continues to enrich, transporting yolk substances, such as VLDLs, through the follicle stalk to the vascular network of the follicle membrane layer. Yolk substances enter the interior of the follicle from the gap between granulosa cells, causing changes in follicle color and volume [14,15]. Therefore, distinguishing between different types of follicles requires a comprehensive consideration of color, diameter, and vascular richness. We considered these multiple factors for follicle classification in our study, and the accuracy of the follicle classification method was reconfirmed through PCA and DEG analysis of sequencing data.

High-throughput sequencing was performed on follicular granulosa cells at the stages of LWFs, SYFs, F5, and F4, and trend and Venn analyses were subsequently conducted on the transcriptome data. The transcripts that remained unchanged in the LWF to SYF and F5 to F4 stages but exhibited significant changes between the SYF to F5 stage were identified as transcripts important for goose follicle selection. Not surprisingly, after enrichment analysis of the selected mRNAs and miRNA and lncRNA, it was found that the extracellular matrix, cell junctions, glucose and lipid metabolism, and signal transduction pathways fluctuated significantly during follicular selection. Another study in geese also found that junctional adhesion and lipid metabolism play crucial roles in follicle selection [8,16], which further corroborates our results.

For this study, the most important thing is not to identify the signaling pathways that change during follicular selection, but to find the transcripts that actively participate in follicular selection activities without hypotheses. Using a new data-screening method, we identified 71 miRNAs that play crucial roles in goose egg follicle selection. Among them, miR-222-3p, miR-2954-3p, miR-126-5p, miR-2478, and miR-425-5p showed high expression levels. Because these miRNAs have not been extensively studied in geese, we can infer their functions from other animal models. Studies in cows have shown that the abundance of miR-222-3p in ovarian granulosa cells is regulated by FSH [17] and may affect ovarian function by regulating LHGCR expression [18]. Other studies suggest that miR-222-3p is involved in the follicular selection process in cattle [19]. In addition, several studies in humans have shown that mir-222-3p is implicated in polycystic ovary syndrome (PCOS), which is related to follicular dysplasia [20,21]. Like miR-222-3p, miR-126-5p is also associated with PCOS [22,23]. Studies in rats and pigs have shown that miR-126-5p in ovarian granulosa cells promotes vascular growth and inhibits apoptosis [24,25,26]. There is limited research on the role of miR-2954 in reproduction. Studies in chickens suggest that miR-2954 plays an important role in gonadal differentiation [27], ovarian cell proliferation and differentiation, steroid hormone synthesis, and angiogenesis [28]. Some studies suggest that miR-425-5p is implicated in human PCOS [29]. Additionally, miR-425-5p is reported to target TGFBR2 to regulate apoptosis in porcine granulosa cells [30]. Research on miR-2478 in cattle highlights significant expression differences between cows with high and low reproductive performance [31]. However, there are no relevant reports on whether miR-2478 is directly involved in the regulation of follicular development. Although all the five key miRNAs screened were implicated in goose follicular selection for the first time, miR-222-3p and miR-126-5p have been repeatedly confirmed to be closely related to follicular development in other species. However, there are no reports on the involvement of miR-2954-3p, miR-2478, or miR-425-5p in animal follicular development or selection. This study provides new targets for studying the regulatory mechanisms of follicular selection, especially in geese. In addition, the specific regulatory mechanism of miRNAs can be inferred from the constructed ceRNA network.

## 5. Conclusions

In conclusion, we confirmed the significant changes in the extracellular matrix, cell junction, and glucose and lipid metabolism during follicular selection. More importantly, using a new analytical method, we identified miRNAs that play an important role in the follicular selection stage. It is worth noting that there are currently no reports on the involvement of miR-2954-3p, miR-2478, or miR-425-5p in follicular selection, which may provide a new perspective for understanding follicular development, especially goose follicular development. The constructed ceRNA network helps to identify the regulatory mechanisms of the selected miRNAs. With the help of high-throughput sequencing, this study has reported for the first time that five miRNAs, including miR-222-3p, miR-2954-3p, miR-126-5p, miR-2478, and miR-425-5p, may play an important role in the follicular selection process of geese and provides potential targets for the precise regulation of goose egg production performance.

## Data Availability

The original contributions presented in this study are included in the article/Supplementary Material, and further inquiries can be brought to the corresponding author.

## Supplementary Materials

The following supporting information can be downloaded at: https://www.mdpi.com/article/10.3390/ani13132132/s1, Table S1: Information list of sequencing data; Table S2: Primer sequences used in this study.
