# Supplementary material for: Transcriptome Profiling of Goose Ovarian Follicle Granulosa Cells Reveals Key Regulatory Networks for Follicle Selection
Source: Animals (Basel). 2023 Jun 28;13(13):2132. doi: 10.3390/ani13132132 (PMC10339945; doi:10.3390/ani13132132)
Supplement: Supplementary file 1 [file animals-13-02132-s001.zip › animals-2428170-supplementary.pdf]

Table S1. Information list of sequencing data.

Sample	Total	Unmapped(%)	Unique_Mapped(%)	Multiple_Mapped(%)	Total_Mapped(%)
LWF-1	1.14E+08	15135544 (13.32%)	95815547 (84.32%)	2681971 (2.36%)	98497518 (86.68%)
LWF-2	74909912	8833500 (11.79%)	64400094 (85.97%)	1676318 (2.24%)	66076412 (88.21%)
LWF-3	1.06E+08	11571779 (10.93%)	91910445 (86.83%)	2367818 (2.24%)	94278263 (89.07%)
SYF-1	1.19E+08	15751555 (13.28%)	100242346 (84.54%)	2579921 (2.18%)	102822267 (86.72%)
SYF-2	1.3E+08	17118186 (13.12%)	110314166 (84.56%)	3022926 (2.32%)	113337092 (86.88%)
SYF-3	89261138	11417183 (12.79%)	75853578 (84.98%)	1990377 (2.23%)	77843955 (87.21%)
F5-1	78051398	8302867 (10.64%)	68607857 (87.90%)	1140674 (1.46%)	69748531 (89.36%)
F5-2	80379448	8160792 (10.15%)	71037405 (88.38%)	1181251 (1.47%)	72218656 (89.85%)
F5-3	93626910	10912165 (11.65%)	81188341 (86.71%)	1526404 (1.63%)	82714745 (88.35%)
F4-1	1.11E+08	13318611 (12.02%)	95554490 (86.20%)	1976185 (1.78%)	97530675 (87.98%)
F4-2	1.1E+08	10884649 (9.90%)	97419707 (88.61%)	1637126 (1.49%)	99056833 (90.10%)
F4-3	1.29E+08	13723305 (10.61%)	113533880 (87.81%)	2041193 (1.58%)	115575073 (89.39%)

Table S2. Primer sequences used in this study.

Primers Name	GeneBank Accession	Sequences (5′-3′)	PCR Products (bp)
FGL2	XM_013180410.2	F: caagcactcccacccatttc	135
		R: aagcagcttcatcttcgcag	
RUSC2	XM_013200524.2	F: gtccataacaagctgctcgg	182
		R: gcgacacgttcacatagacc	
BSDC1	XM_048049602	F: accacagaaccctacgacag	152
		R: tccgcaatctcccctttctt	
XR_001208580.1	XR_001208580.1	F: gggagtcagtcttcaccgaa	87
		R: cattgcggacatctcactgg	
XR_001210752.1	XR_001210752.1	F: cagagtgccacgatgcttac	141
		R: attttcaggggaggggatgg	
XR_001213437.1	XR_001213437.1	F: tgccttactccgtgaacagt	100
		R: tctcctcttgtgaagcagca	
γ-DH	NM_204305.1	F:gccatcacagccacacaga	120
		R: tttccccacagccttagca	
